# Pubic Arch Angle Measurement by Transperineal Ultrasonography: A Prospective Cross-Sectional Study

**DOI:** 10.1055/s-0040-1709690

**Published:** 2020-04

**Authors:** Raimundo Homero Carvalho Neto, Antonio Brazil Viana Junior, Antonio Fernandes Moron, Edward Araujo Júnior, Francisco Herlânio Costa Carvalho, Helvécio Neves Feitosa

**Affiliations:** 1 Maternal Fetal Medicine Service, Maternidade Assis Chateaubriand, Universidade Federal do Ceará, Fortaleza, CE, Brazil; 2 Department of Obstetrics, Escola Paulista de Medicina, Universidade Federal de São Paulo, São Paulo, SP, Brazil; 3 Medical Course, Universidade Municipal de São Caetano do Sul, São Paulo, SP, Brazil; 4 Department of Obstetrics and Gynecology, Universidade de Fortaleza, Fortaleza, CE, Brazil

**Keywords:** labor, ultrasonography, cephalopelvic disproportion, vaginal delivery, cesarean section, trabalho de parto, ultrassonografia, desproporção cefalo-pélvica, parto vaginal, cesárea

## Abstract

**Objective** To evaluate the ability of the pubic arch angle (PAA) as measured by transperineal ultrasonography during labor to predict the delivery type and cephalic pole disengagement mode.

**Methods** The present prospective cross-sectional study included 221 women in singleton-gestational labor ≥ 37 weeks with cephalic fetuses who underwent PAA measurement using transperineal ultrasonography. These measurements were correlated with the delivery type, cephalic pole disengagement mode, and fetal and maternal characteristics.

**Results** Out of the subjects, 153 (69.2%) had spontaneous vaginal delivery, 7 (3.2%) gave birth by forceps, and 61 (27.6%) delivered by cesarean section. For the analysis, deliveries were divided into two groups: vaginal and surgical (forceps and cesarean). The mean PAA was 102 ± 7.5° (range, 79.3–117.7°). No statistically significant difference was observed in delivery type (102.6 ± 7.2° versus 100.8 ± 7.9°, *p* = 0.105). The occipitoanterior position was seen in 94.1% of the fetuses and the occipitoposterior position in 5.8%. A narrower PAA was found in the group of surgical deliveries (97.9 ± 9.6° versus 102.6 ± 7.3°, *p* = 0.049). Multivariate regression analysis showed that PAA was a predictive variable for the occurrence of head disengagement in occipital varieties after birth (odds ratio, 0.9; 95% confidence interval, 0.82–0.99; *p* = 0.026).

**Conclusion** Ultrasonographic measurement of the PAA was not a predictor of delivery type, but was associated with the persistence of occipital varieties after birth.

## Resumo

**Objetivo** Avaliar a medida do ângulo do arco púbico (AAP) por ultrassonografia transperineal durante trabalho de parto em predizer tipo de parto e modo de desprendimento do polo cefálico.

**Métodos** Um estudo prospectivo transversal foi conduzido com 221 mulheres em trabalho de parto com gestação única ≥ 37 semanas, com fetos em apresentação cefálica, foram submetidas à avaliação ultrassonográfica por via transperineal para aferição do AAP. Correlações com tipo de parto, modo de desprendimento do polo cefálico e características fetais e maternas foram realizadas.

**Resultados** Um total de 153 (69,2%) mulheres apresentaram parto vaginal espontâneo, 7 (3,2%) parto a fórceps e 61 (27,6%) parto cesárea. Para fins de análise, dividiu-se os partos em dois grupos: partos vaginais e cirúrgicos (fórceps e cesáreas). A média do AAP foi 102 ± 7,5° (variação: 79,3–117,7°). Não foi observada significância estatística do AAP em relação ao tipo de parto (102,6 ± 7,2° versus 100,8 ± 7,9°; *p* = 0,105). Um total de 94,1% dos fetos desprenderam em variedade de posição occipito anterior e 5,8% em occipito posterior. Encontrou-se AAP mais estreitado no grupo de partos cirúrgicos (97,9 ± 9,6° *versus* 102,6 ± 7,3°; *p* = 0,049). A análise de regressão multivariada demonstrou que AAP foi uma variável de proteção para a ocorrência de desprendimento da cabeça em variedades occipito posteriores ao nascimento (odds ratio [OR]= 0,9; índice de confiança (IC) 95%: 0,82–0,99; *p* = 0,026).

**Conclusão** A medida ultrassonográfica do AAP não foi preditora do tipo de parto, porém demonstrou associação com persistência de variedades occipito posteriores ao nascimento.

## Introduction

A good proportion between the fetal head and maternal pelvis is a fundamental condition for the physiological presentation of childbirth. During its descent, the cephalic pole performs flexion, rotation, and extension and develops plastic alterations in its format. The birth canal also adapts—that is, mobility of the sacrococcygeal joint increases and the soft tissues become distended. Such changes are necessary since the head diameters of a term fetus are similar to the main diameters of the pelvis, requiring the latter to adapt to the birth canal to enable the fetus to cross it.^1^

The disparity between pelvic architecture or size and the fetal head constitutes an obstetric entity called cephalopelvic disproportion (CPD), a cause of increased operative emergencies during delivery and adverse perinatal outcomes, accounting for 8% of all maternal deaths worldwide.^2^ Cephalopelvic disproportion is diagnosed during labor, and its prediction at the end of gestation or onset of labor improves fetal outcomes and avoids stress and dissatisfaction in pregnant women due to prolonged labor that ultimately results in emergency cesarean section.^3^

Pelvimetry, a method that studies pelvic shape and proportions, can be performed clinically through the measurement of the diagonal conjugate, interischial distance, and bituberous diameter^1^ or using imaging methods such as radiography, computed tomography (CT), magnetic resonance imaging (MRI), and ultrasonography.^4^ ^5^ The accuracy of the clinical detection of pelvic narrowing is limited to 50%.^6^ The use of X-rays not only causes exposure to ionizing radiation, but also doubles the incidence of abdominal births.^7^ Computed tomography and MRI are effective but very costly and often impractical within an obstetric center.

Ultrasonography is highly accessible in delivery rooms, has a smaller learning curve, is painless, is easy to perform, and is relatively innocuous. For these reasons, it has become a widely used method.^8^ ^9^ ^10^ ^11^ ^12^ The main parameter studied on ultrasonography in such cases is the pubic arch angle (PAA), which is formed by the confluence of the pubic bone rami at the level of the symphysis.^13^ This angle provides indirect information about pelvic shape and obstetric dimensions such as the superior aperture of the pelvis and the interspinous distance.^14^ The gynecoid pelvis has a wide PAA and favors rotation of the cephalic pole to the occipitoanterior position. In women with a narrow anterior pelvic compartment in which the PAA is decreased, as in android pelvis, the pubic rami converge at a sharper angle. In these situations, the fetal head tends to position itself in the posterior compartment of the birth canal, being forced against the soft tissues and bony structures in this region. This impairs the rotation of the occiput to the anterior positions, increasing the frequency of transversal and persistent posterior varieties and leading to the occurrence of dystocia and surgical delivery.^15^ ^16^ ^17^

Several studies have evaluated the efficacy of the PAA measurement, both before and during labor, at predicting the delivery route and cephalic pole detachment mode.^13^ ^17^ These studies examined specific population groups from Europe, the Middle East, and Oceania. However, evaluations in other populations with different anthropometric characteristics are required to corroborate the applicability of this method and increase its acceptability; notably in the Brazilian population that presents anthropometric heterogeneity due to its racial mixture.

The objective of the present study was to analyze whether the PAA measure, as a parameter of pelvic proportion, is able to predict the delivery type and cephalic pole disengagement mode.

## Methods

The present prospective cross-sectional study was conducted between February and September 2017 at the Assis Chateaubriand Teaching Maternity of the Universidade Federal do Ceará (UFC), Fortaleza, state of Ceará, Brazil. A convenience sample of 221 parturients was recruited in the first or second phase of labor according to the clinical evolution at admission. Transperineal ultrasonography was used to measure the PAA (exposure variable); these data were compared with delivery type (vaginal and surgical) and cephalic pole disengagement mode (occipitoanterior or occipitoposterior, variables of outcome) in search of associations. The sum of the forceps and cesarean deliveries was considered surgical delivery. Other relevant information possibly capable of predicting delivery type and cephalic pole disengagement mode or of distorting the associations described above was also studied. This included maternal age, maternal height, body mass index (BMI), parity, birthweight, type of labor onset (spontaneous or induced), labor analgesia, and use of uterotonic agents. Gestational age was not compared because all patients in the study were full term (≥ 37 weeks). Informed consent was obtained from all patients, and the present study was approved by the UFC Research Ethics Committee under the opinion number 1.010.040.

The inclusion criterion was a singleton pregnancy with a live fetus without structural anomalies in cephalic presentation with estimated fetal weight by ultrasound considered adequate for gestational age and biparietal diameter < 2 standard deviations (SDs) for gestational age; regardless of whether the amniotic sac was intact or broken or whether the patients received labor analgesia. Patients were excluded if on admission they presented urgent situations requiring immediate pregnancy resolution by cesarean section, such as: uterine rupture, umbilical cord prolapse, placental abruption with changes in fetal auscultation, and cardiotocographic tracings classified in category 3 of the National Institute of Child Health and Human Development 2008.^18^ Also, newborns with ≥ 4,000 g were excluded.

Pubic arch angle measurement was performed by a single examiner (Carvalho R. H.) using a Logic C5 Premium ultrasound device (General Electric, Milwaukee, WI, USA) equipped with a two-dimensional (3–5 MHz) convex transducer. The PAA measurements were obtained transperineally, outside the period of contraction or pull, with the women in the dorsal decubitus position and the legs ajar and semi-flexed.

**Fig. 1:**
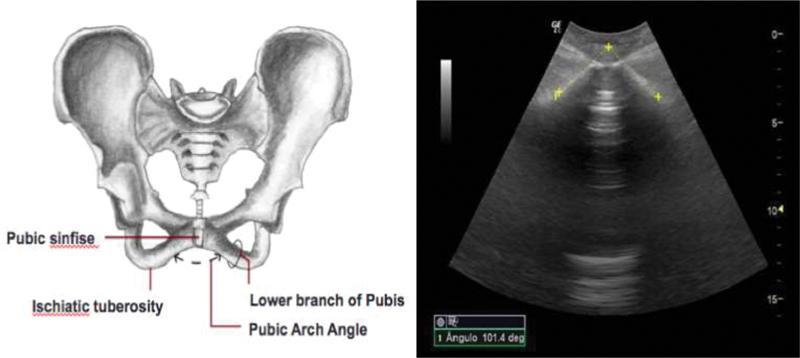
Schematic drawing of the pubic arch angle (PAA) (left) and PAA image obtained by transperineal ultrasonography during the first phase of delivery (right).

The probe was positioned transversally in contact with the perineum at the level of the clitoris. The transducer was tilted at ∼ 45° until an image of the symphysis with the 2 branches of the pubic bone in symmetrical position was obtained. The lines for angle measurement were positioned on the lower edges of the right and left pubic branches, forming a triangle based on the ischial tuberosities bilaterally and on the convergence at the center of the symphysis as the apex (Fig. 1), as previously described by Gilboa et al.^13^ Three PAA measurements were obtained from each participant, and the average of the three measurements was considered.

Ultrasound findings were not revealed to the members of the obstetrical staff to avoid interference with labor. The follow-up of the delivery was the responsibility of the on-call care team, which followed the routine recommended by institutional protocols.^19^

The descriptive data are presented as mean ± SD or *n* (%). The Chi-squared and Fisher exact tests were used to analyze the categorical variables, and the Mann-Whitney or Student *t*-test test was used to analyze continuous variables according to the normality of the data. The analyzed variables were PAA; patient age, height, body mass index (BMI), and parity; birth weight; labor onset type (spontaneous or induced); labor analgesia; and the use of uterotonic agents according to delivery type (vaginal or surgical) and cephalic pole disengagement mode (occipitoanterior or occipitoposterior) as the outcome variables.

All variables presenting a value of *p* < 0.20 for one or both outcomes were subjected to multiple logistic regression analysis for both outcomes (surgical delivery and cephalic pole disengagement in the occipitoposterior position). Values of *p* < 0.05 were considered statistically significant. The gross odds ratio (OR) was calculated and adjusted with its respective 95% confidence interval (CI). The data were analyzed by SPSS for Windows, version 13.0 (SPSS, Inc., Chicago, IL, USA).

## Results

The clinical and obstetric characteristics of the 221 participants as well as the birth and outcome details are shown in Table 1.

**Table 1:** Characteristics of the study population, deliveries, and outcomes

Variable	Mean ± SD	*n* (%)
Maternal age (years old)	24.2 ± 6.8	–
Parity		
0	–	129 (58.4)
1	–	63 (28.5)
2	–	19 (8.6)
3	–	7 (3.2)
4	–	1 (0.5)
5	–	1 (0.5)
9	–	1 (0.5)
Maternal height (m)	1.57 ± 0.6	–
Maternal weight (kg)	72.4 ± 14	–
BMI (kg/m^2^)	29.3 ± 4.7	–
Gestational age (US) (weeks)	39.4 ± 1.1	–
Birth weight (g)	3.312.1 ± 427	–
Labor analgesia	–	32 (14.5)
Use of uterotonic agents	–	78 (36.1)
Previous cesarean section	–	16 (7.2)
Labor induction	–	16 (7.3)
Delivery type		
Spontaneous vaginal	–	153 (69.2)
Forceps	–	7 (3.2)
Cesarean section	–	61 (27.6)
Cephalic pole disengagement		
Occipitoanterior	–	161 (94.2)
Occipitoposterior	–	10 (5.8)

Abbreviations: BMI, body mass index; SD, standard deviation; US, ultrasonography.

There were 153 (69.2%) vaginal deliveries, 7 (3.2%) forceps deliveries, and 61 (27.6%) cesarean deliveries. Table 2 shows the univariate analysis results stratified according to delivery type (vaginal versus surgical). Surgical delivery was associated with shorter maternal height (1.58 ± 0.06 m versus 1.55 ± 0.06 m; *p* < 0.001), higher BMI (28.6 ± 4.6 kg/m^2^ versus 30.8 ± 4.7 kg/m^2^; *p* < 0.001), greater use of labor analgesia (15/153 or 9.9% versus 17/68 or 17%; *p* < 0.003), and lower parity (78/153 or 51% versus 51/68 or 75%; *p* < 0.001). No statistically significant difference was observed for PAA among delivery types (102.6 ± 7.20° versus 100.8 ± 7.90°; *p* = 0.105).

**Table 2:** Population characteristics stratified by delivery type

	Vaginal (*n* = 153)	Surgical (forceps and cesarean section) (*n* = 68)	*p-value*
Age (years old)	24.0 ± 6.91	24.57 ± 6.66	0.588^a^
Height (m)	1.58 ± 0.06	1.55 ± 0.06	< 0.001^a^
BMI (kg/m^2^)	28.6 ± 4.55	30.82 ± 4.73	0.001^a^
Parity			0.001^b^
Nulliparous	78 (51%)	51 (75%)	
Multiparous	75 (49%)	17 (25%)	
Labor analgesia	15 (9.9%)	17 (25%)	< 0.003^b^
Delivery type			0.622^b^
Spontaneous	141 (92.2%)	63 (94%)	
Induced	12 (7.8%)	4 (6%)	
Use of uterotonic agents	48 (32.2%)	30 (44.8%)	0.075^b^
Birth weight (g)	3287.9 ± 429.8	3366.4 ± 418.84	0.208^a^
Pubic arc angle (°)	102.56 ± 7.22	100.8 ± 7.96	0.105^a^

Abbreviation: BMI, body mass index.

a Student *t*-test.

b Chi-squared test.

Table 3 shows the stratification of the data by fetal occiput position on disengagement. In 171/221 study patients, it was possible to retrieve this information from the medical records. An association was noted between the use of labor analgesia and the occurrence of disengagement in the occipital position at birth (20/161 or 12.2% versus 4/10 or 40%; *p* = 0.013). Pubic arch angle regarding fetal occiput position on disengagement differed significantly. Narrower angles were associated with occipitoposterior positions (102.6 ± 7.30° versus 97.9 ± 9.60°; *p* = 0.049).

**Table 3:** Population characteristics stratified by fetal occiput position on disengagement

Variable	Occipitoanterior (*n* = 161)	Occipitoposterior (*n* = 10)	*p-value*
Age (years old)	24.17 ± 6.71	22 ± 7.24	0.324^a^
Height (m)	1.58 ± 0.06	1.58 ± 0.05	0.647^a^
BMI (kg/m^2^)	28.95 ± 4.61	27.8 ± 3.54	0.444^a^
Parity			0.271^b^
Nulliparous	86 (52.1%)	7 (70%)	
Multiparous	79 (47.9%)	3 (30%)	
Labor analgesia	20 (12.2%)	4 (40%)	0.013^b^
Delivery type			0.670^b^
Spontaneous	153 (92.7%)	8 (88.9%)	
Induced	12 (7.3%)	1 (11.1%)	
Use of uterotonic agents	53 (32.9%)	6 (60%)	0.080^b^
Birthweight (g)	3310.38 ± 430.27	3088 ± 296.41	0.109^a^
Pubic arc angle (°)	102.58 ± 7.27	97.92 ± 9.59	0.049^a^

Abbreviation: BMI, body mass index.

a Student *t*-test.

b Chi-squared test.

The results of the multivariate regression model of variables with *p* values < 0.20 are shown in Tables 4 and 5. In the analysis of the surgical delivery type outcome (Table 4), 4 variables were relevant, representing a risk for this type of resolution: maternal height < 1.57 m (OR: 3.05; 95%CI: 1.55–6.02; *p* = 0.001); BMI/obesity (OR: 3.89; 95%CI: 1.38–10.93; *p* = 0.010); nulliparity (OR: 2.89; 95%CI: 1.42–5.87; *p* = 0.003), and use of labor analgesia (OR: 2.68; 95%CI: 1.08–6.68; *p* = 0.034). When analyzing the outcome variable of cephalic pole disengagement in the posterior positions (Table 5), labor analgesia did not differ significantly, unlike the PAA, which showed an association as a protection factor for this occurrence (OR: 0.90; 95%CI: 0.82–0.99; *p* = 0.026). The PAA showed a negative correlation with fetal head disengagement at the occipitoposterior position — that is, each degree of PAA decrease caused an 11% increase in the risk of delivery with the cephalic pole at the posterior occipitoposition.

**Table 4:** Logistic regression analysis results of surgical delivery outcome

Variable	Univariate			Multivariate		
	B	OR (95%CI)	*p-value*	B	OR (95%CI)	*p-value*
Height < 1.57 m	0.95	2.58 (1.41–4.73)	0.002	1.12	3.05 (1.55–6.02)	0.001
BMI (kg/m^2^)						
Adequate		reference				
Overweight	0.87	2.38 (1.03–7.293)	0.043	0.90	2.47 (0.87–6.97)	0.089
Obese	0.34	3.62 (1.39–9.46)	0.009	1.36	3.89 (1.38–10.93)	0.010
Nulliparous	1.06	2.89 (1.53–5.44)	0.001	1.06	2.89 (1.42–5.87)	0.003
Labor analgesia	1.11	3.04 (1.42–6.54)	0.004	0.99	2.68 (1.08–6.68)	0.034
Use of uterotonic agents	0.53	1.71 (0.94–3.08)	0.007	0.21	1.22 (0.60–2.51)	0.574
Birth weight > 3,325 g	0.27	1.31 (0.74–2.32)	0.358	0.40	1.5 (0.77–2.92)	0.236
Pubic arc angle (°)	-0.03	0.97 (0.93–1.01)	0.106	-0.04	0.97 (0.92–1.01)	0.116

B, variable coefficient in the regression model; BMI, body mass index; CI, confidence interval; OR, odds ratio.

R^2^ Nagelkerke = 0.24; Median height, 1.57 m (1.54–1.1 m); Median birthweight, 3,325 g (2,995–3,615 g).

**Table 5:** Logistic regression analysis results of fetal head disengagement (occipitoposterior) outcome

Variable	Univariate			Multivariate		
	B	OR (95%CI)	*p-value*	B	OR (95%CI)	*p-value*
Height < 1.57 m	0.04	1.04 (0.29–3.72)	0.955	-0.08	0.92 (0.22–3.86)	0.913
BMI (kg/m^2^)						
Adequate		reference				
Overweight	0.11	1.11 (0.26–4.72)	0.886	0.23	1.25 (0.25–6.19)	0.782
Obese	−1.75	0.17 (0.02–1.74)	0.136	−2.01	0.13 (0.01–1.56)	0.108
Nulliparous	0.76	2.14 (0.54–8.58)	0.281	0.28	1.32 (0.26–6.80)	0.737
Labor analgesia	1.57	4.8 (1.25–18.49)	0.023	1.08	2.96 (0.56–15.71)	0.203
Use of uterotonic agents	0.53	1.71 (0.94–3.08)	0.094	0.79	2.20 (0.46–10.43)	0.320
Birth weight > 3325 g	−1.37	0.25 (0.05–1.23)	0.088	−1.45	0.24 (0.04–1.50)	0.126
Pubic arc angle (°)	−0.08	0.93 (0.86–1)	0.060	−0.11	0.90 (0.82–0.99)	0.026

B, variable coefficient in the regression model; BMI, body mass index; CI, confidence interval; OR, odds ratio.

R^2^ Nagelkerke = 0.274; Median height, 1.57 m (1.54–1.1 m); Median birth weight, 3,325 g (2,995–3,615 m).

## Discussion

Most groups that use intrapartum ultrasound in centers in Europe, the Middle East, Asia, and North America apply three-dimensional (3D) technology.^13^ ^15^ ^17^ ^20^ In the present study, two-dimensional (2D) ultrasonography was used since it is the method available in most maternity hospitals in Brazil and is less costly and easier to perform. Torkildsen et al^21^ found good intraobserver agreement and reproducibility between 2D and 3D techniques. Corroborating this finding, the results of the present study were close to those found in studies that used 2D technology, with a mean PAA of 102.0 ± 7.5°. Using 2D ultrasonography, Gilboa et al^13^ found a mean PAA of 101.1 ± 13.1° in a cohort of 62 Israeli women in the prolonged second stage of labor. Applying 3D technology, Albrich et al^20^ found a mean PAA of 109.3 ± 8.9° in a cohort of 611 Australian women at between 34 and 36 weeks of gestation.

Our study results demonstrate that maternal height, BMI, and epidural analgesia influence delivery type. Surgical delivery occurred more frequently in shorter or obese women as well as in those who used labor analgesia, corroborating findings in the literature, which demonstrated an increase in the incidence of surgical delivery with short maternal height and obesity^22^ ^23^ as well as higher occurrences of instrumental deliveries in patients receiving epidural analgesia.^24^

The PAA was not a predictor of delivery type as in other studies, which also failed to demonstrate this association.^20^ ^25^ However, contrary results were obtained by Gilboa et al^13^ and Ghi et al,^17^ who observed that women with surgical delivery outcomes had smaller PAAs than those who had vaginal delivery (97.1 ± 11.5° versus 110.1 ± 14.0° and 111.4 ± 13.5° versus 118.4 ± 11.4°, respectively). It should be considered that, unlike in the current study, the population evaluated in those studies consisted only of pregnant women in the second phase of labor;^13^ ^17^ in one, all patients selected presented progression failure at this stage.^13^ Some factors should be considered in the evaluation of these conflicting results, such as different study designs, various pelvic conformations, and various delivery modes and different rates of uterotonic agent use and analgesia that affect the local incidence of cesarean section and interfere with the study findings.

Considering the fetal head disengagement outcome variable, the PAA was associated with the occurrence of occipitoposterior varieties. Smaller PAAs were observed in patients who delivered fetuses in the occipitoposterior position than in the anterior positions. Ghi et al^17^ also found an association between PAA narrowing and the occipitoposterior variety at delivery (OR: 1.04; 95%CI: 1.01–1.08). These results demonstrated a lower PAA in patients who gave birth to fetuses with heads in posterior varieties than in those whose fetuses were delivered in anterior positions (104.3 ± 16.8° versus 116.4 ± 11.9°).^17^ The findings of these two studies reinforce a recent hypothesis in the literature that assumes that the occurrence of persistent occipitoposterior varieties in labor may be an adaptive phenomenon to narrowing of the anterior pelvic compartment.^15^ When the care team is aware that the parturient has a reduced PAA, this provides guidance on a likely prolongation of labor in addition to favoring a more attentive attitude regarding the possibility of dystocia as well as the possible need for an instrumental delivery and an episiotomy.

Regarding the limitations of the present study, it is possible that the low socioeconomic level of the studied population impacted the different types of childbirth. The preference for cesarean section influenced by sociocultural factors^26^ was associated with the low use of instrumental delivery and the nonuse of manual head rotation in the facility where the present study was performed, resulting in an increased incidence of cesarean section, and may have interfered with the attempt to demonstrate the association of the PAA with delivery type. No other methods, such as CT or MRI, were used to validate the ultrasound measurements. The incomplete medical records caused gaps that made it difficult to analyze some of the data more substantially, such as the duration of the second phase of labor that was cited by Gilboa et al^13^ as inversely proportional to the measurement of the PAA. Another limitation to be considered is that the present study did not intend to apply 3D ultrasound or other 2D ultrasound parameters to assess maternal pelvis or fetal head malposition (deflection and asynclitism) that contribute to the occurrence of dystocia.^27^

New studies are required to clarify the discordant results in the literature regarding the influence of PAA on the evolution of labor. However, the importance of this knowledge for better delivery assistance is well understood at our institution; due to the good sampling and technical rigor used, these data can be extrapolated to parturients similar to those included in the present study.

## Conclusion

In summary, the ultrasound measurement of the PAA was not a predictor of delivery type but was associated with the persistence of occipitoposterior varieties in fetal head disengagement.

## References

[1] Neal J L Lowe N K Caughey A B Bennet K A Tilden E L Carlson N S Applying a physiologic partograph to Consortium on Safe Labor data to identify opportunities for safely decreasing cesarean births among nulliparous women Birth 2018 45 04 358 367. Doi: 10.1111/birt.12358 29851163 10.1111/birt.12358 PMC6342020

[2] Filippi V Chou D Ronsmans C Graham W Say L Levels and causes of maternal mortality and morbidity Washington (DC) The International Bank for Reconstruction and Development/The World Bank 2016. vol. 2, p. 51–70 27227230

[3] Yeo L Romero R Sonographic evaluation in the second stage of labor to improve the assessment of labor progress and its outcome Ultrasound Obstet Gynecol 2009 33 03 253 258. Doi: 10.1002/uog.6336 19247999 10.1002/uog.6336 PMC3138397

[4] Suramo I Torniainen P Jouppila P Kirkinen P Lähde S A low-dose CT-pelvimetry Br J Radiol 1984 57 673 35 37. Doi: 10.1259/0007-1285-57-673-35 6704646 10.1259/0007-1285-57-673-35

[5] Spörri S Hänggi W Braghetti A Vock P Schneider H Pelvimetry by magnetic resonance imaging as a diagnostic tool to evaluate dystocia Obstet Gynecol 1997 89 06 902 908. Doi: 10.1016/s0029-7844(97)00148-8 9170462 10.1016/s0029-7844(97)00148-8

[6] Dudding T C Vaizey C J Kamm M A Obstetric anal sphincter injury: incidence, risk factors, and management Ann Surg 2008 247 02 224 237. Doi: 10.1097/SLA.0b013e318142cdf4 18216527 10.1097/SLA.0b013e318142cdf4

[7] Pattinson R C Pelvimetry for fetal cephalic presentations at term Cochrane Database Syst Rev 2000 02 CD000161. Doi: 10.1002/14651858.CD000161 10796162 10.1002/14651858.CD000161

[8] Sherer D M Miodovnik M Bradley K S Langer O Intrapartum fetal head position II: comparison between transvaginal digital examination and transabdominal ultrasound assessment during the second stage of labor Ultrasound Obstet Gynecol 2002 19 03 264 268. Doi: 10.1046/j.1469-0705.2002.00656.x 11896948 10.1046/j.1469-0705.2002.00656.x

[9] Akmal S Kametas N Tsoi E Hargreaves C Nicolaides K H Comparison of transvaginal digital examination with intrapartum sonography to determine fetal head position before instrumental delivery Ultrasound Obstet Gynecol 2003 21 05 437 440. Doi: 10.1002/uog.103 12768552 10.1002/uog.103

[10] Chou M R Kreiser D Taslimi M M Druzin M L El-Sayed Y Y Vaginal versus ultrasound examination of fetal occiput position during the second stage of labor Am J Obstet Gynecol 2004 191 02 521 524. Doi: 10.1016/j.ajog.2004.01.029 15343230 10.1016/j.ajog.2004.01.029

[11] Rozenberg P Porcher R Salomon L J Boirot F Morin C Ville Y Comparison of the learning curves of digital examination and transabdominal sonography for the determination of fetal head position during labor Ultrasound Obstet Gynecol 2008 31 03 332 337. Doi: 10.1002/uog.5267 18307213 10.1002/uog.5267

[12] Barbera A F Pombar X Perugino G Lezotte D C Hobbins J C A new method to assess fetal head descent in labor with transperineal ultrasound Ultrasound Obstet Gynecol 2009 33 03 313 319. Doi: 10.1002/uog.6329 19248000 10.1002/uog.6329

[13] Gilboa Y Kivilevitch Z Spira M Kedem A Katorza E Moran O Achiron R Pubic arch angle in prolonged second stage of labor: clinical significance Ultrasound Obstet Gynecol 2013 41 04 442 446. Doi: 10.1002/uog.12304 23001876 10.1002/uog.12304

[14] Yeomans E R Clinical pelvimetry Clin Obstet Gynecol 2006 49 01 140 146. Doi: 10.1097/01.grf.0000198185.94413.0d 16456351 10.1097/01.grf.0000198185.94413.0d

[15] Benavides L Wu J M Hundley A F Ivester T S Visco A G The impact of occiput posterior fetal head position on the risk of anal sphincter injury in forceps-assisted vaginal deliveries Am J Obstet Gynecol 2005 192 05 1702 1706. Doi: 10.1016/j.ajog.2004.11.047 15902181 10.1016/j.ajog.2004.11.047

[16] Caughey A B Sharshiner R Cheng Y W Fetal malposition: impact and management Clin Obstet Gynecol 2015 58 02 241 245. Doi: 10.1097/GRF.0000000000000106 25851845 10.1097/GRF.0000000000000106

[17] Ghi T Youssef A Martelli F Bellussi F Aiello E Pilu G Narrow subpubic arch angle is associated with higher risk of persistent occiput posterior position at delivery Ultrasound Obstet Gynecol 2016 48 04 511 515. Doi: 10.1002/uog.15808 26565728 10.1002/uog.15808

[18] Macones G A Hankins G DV Spong C Y Hauth J Moore T The 2008 National Institute of Child Health and Human Development workshop report on electronic fetal monitoring: update on definitions, interpretation, and research guidelines Obstet Gynecol 2008 112 03 661 666. Doi: 10.1097/AOG.0b013e3181841395 18757666 10.1097/AOG.0b013e3181841395

[19] Universidade Federal do Ceará. Maternidade Escola Assis Chateaubriand. Protocolos e diretrizes terapêuticas: Unidade 6- obstetrícia [Internet] 2017 [cited 2018 Jan 05]. Available from: http://www.ebserh.gov.br/web/meac-ufc/protocolos-e-pops

[20] Albrich S B Shek K Krahn U Dietz H P Measurement of subpubic arch angle by three-dimensional transperineal ultrasound and impact on vaginal delivery Ultrasound Obstet Gynecol 2015 46 04 496 500. Doi: 10.1002/uog.14814 25678020 10.1002/uog.14814

[21] Torkildsen E A Salvesen KÅ Eggebø T M Agreement between two- and three-dimensional transperineal ultrasound methods in assessing fetal head descent in the first stage of labor Ultrasound Obstet Gynecol 2012 39 03 310 315. Doi: 10.1002/uog.9065 21630362 10.1002/uog.9065

[22] Toh-Adam R Srisupundit K Tongsong T Short stature as an independent risk factor for cephalopelvic disproportion in a country of relatively small-sized mothers Arch Gynecol Obstet 2012 285 06 1513 1516. Doi: 10.1007/s00404-011-2168-3 22187064 10.1007/s00404-011-2168-3 PMC3351595

[23] Burke N Burke G Breathnach F McAuliffe F Morrison J J Turner M Prediction of cesarean delivery in the term nulliparous woman: results from the prospective, multicenter Genesis study Am J Obstet Gynecol 2017 216 06 5980 5.98E13. Doi: 10.1016/j.ajog.2017.02.017 10.1016/j.ajog.2017.02.017 28213060

[24] Anim-Somuah M Smyth R M Jones L Epidural versus non-epidural or no analgesia in labour Cochrane Database Syst Rev 2011 12 CD000331. Doi: 10.1002/14651858.CD000331.pub3 22161362 10.1002/14651858.CD000331.pub3

[25] Albrich S Laterza R M Merinsky A Skala C Koelbl H Naumann G [Measurement of the infrapubic angle using 3D perineal ultrasound and its relationship to obstetrical parameters] Ultraschall Med 2012 33 07 E95 E100. Doi: 10.1055/s-0031-1299053 22723036 10.1055/s-0031-1299053

[26] Leão M RC Riesco M LG Schneck C A Angelo M [Reflections on the excessive rates of cesareans in Brazil and the empowerment of women] Cien Saude Colet 2013 18 08 2395 2400. Doi: 10.1590/s1413-81232013000800024 23896922 10.1590/s1413-81232013000800024

[27] Bellussi F Ghi T Youssef A The use of intrapartum ultrasound to diagnose malpositions and cephalic malpresentations Am J Obstet Gynecol 2017 217 06 633 641. Doi: 10.1016/j.ajog.2017.07.025 28743440 10.1016/j.ajog.2017.07.025

